# Effect of addition of γ-poly glutamic acid on bacterial nanocellulose production under agitated culture conditions

**DOI:** 10.1186/s13068-024-02515-3

**Published:** 2024-05-27

**Authors:** Yang Bai, Ran Tan, Yiran Yan, Tao Chen, Yetong Feng, Qiwei Sun, Jiakun Li, Yifei Wang, Futao Liu, Jingwen Wang, Yao Zhang, Xianhao Cheng, Guochao Wu

**Affiliations:** 1 Shandong Key Laboratory of Edible Mushroom Technology, School of Agriculture, Ludong University, Yantai, 264025 China; 2 Key Laboratory of Molecular Module-Based Breeding of High Yield and Abiotic Resistant Plants in Universities of Shandong, School of Agriculture, Ludong University, Yantai, 264025 China; 3 School of Chemistry and Materials Science, Ludong University, Yantai, 264025 China

**Keywords:** Bacterial nanocellulose, γ-Polyglutamic acid, Agitated culture, Conversion rate

## Abstract

**Background:**

Bacterial nanocellulose (BNC), a natural polymer material, gained significant popularity among researchers and industry. It has great potential in areas, such as textile manufacturing, fiber-based paper, and packaging products, food industry, biomedical materials, and advanced functional bionanocomposites. The main current fermentation methods for BNC involved static culture, as the agitated culture methods had lower raw material conversion rates and resulted in non-uniform product formation. Currently, studies have shown that the production of BNC can be enhanced by incorporating specific additives into the culture medium. These additives included organic acids or polysaccharides. γ-Polyglutamic acid (γ-PGA), known for its high polymerization, excellent biodegradability, and environmental friendliness, has found extensive application in various industries including daily chemicals, medicine, food, and agriculture.

**Results:**

In this particular study, 0.15 g/L of γ-PGA was incorporated as a medium additive to cultivate BNC under agitated culture conditions of 120 rpm and 30 ℃. The BNC production increased remarkably by 209% in the medium with 0.15 g/L γ-PGA and initial pH of 5.0 compared to that in the standard medium, and BNC production increased by 7.3% in the medium with 0.06 g/L γ-PGA. The addition of γ-PGA as a medium additive resulted in significant improvements in BNC production. Similarly, at initial pH levels of 4.0 and 6.0, the BNC production also increased by 39.3% and 102.3%, respectively. To assess the characteristics of the BNC products, scanning electron microscopy, Fourier transform infrared spectroscopy, and thermogravimetric analysis were used. The average diameter of BNC fibers, which was prepared from the medium adding 0.15 g/L γ-PGA, was twice thicker than that of BNC fibers prepared from the control culture medium. That might be because that polyglutamic acid relieved the BNC synthesis from the shear stress from the agitation.

**Conclusions:**

This experiment held great significance as it explored the use of a novel medium additive, γ-PGA, to improve the production and the glucose conversion rate in BNC fermentation. And the BNC fibers became thicker, with better thermal stability, higher crystallinity, and higher degree of polymerization (DPv). These findings lay a solid foundation for future large-scale fermentation production of BNC using bioreactors.

## Introduction

Bacterial nanocellulose (BNC) is a bio-based polymeric material synthesized mainly by acetic acid bacteria. It has a chemical structure similar to plant cellulose, consisting of straight chain polymers of unbranched macromolecules formed from pyranose monomers (β-D-glucans) linked by β-1,4-glycosidic bonds. BNC has a higher degree of polymerization and crystallinity than plant cellulose, with a fiber diameter of 20–80 nm, which is smaller than that of natural or artificial fibers, and its fermentation process is significantly simpler compared to the methods used for extracting cellulose fibers from plant lignin. The genus of the bacteria which can synthesize nano-bacterial cellulose included *Komagataeibacter, Agrobacterium*, and *Rhizobium*, etc. Among them, the strains of *Komagataeibacter xylinum* had strong extracellular cellulose secretion ability and relatively large bacterial nanocellulose production, which was suitable for large-scale production and application in industry [1, 2].

BNC has a nanofibrillar structure, a high degree of polymerization (DP), and high mechanical strength. These properties enable BNC to have great potential in areas, such as food industry, biomedical applications, papermaking, and non-woven fabrics. In recent years, many breakthroughs on BNC application had been made in the field of biomedicine, mainly involving artificial blood vessels, bionic materials, heart valves, implanted stents, etc. [3–6].

The static fermentation of BNC normally took around 9 d of cultivation [7, 8], and the productivity was also normally low. BNC production was found to be proportional to the surface area of the medium, and was relatively unaffected by the depth and volume of the medium [9]. The membrane grown in static culture prevented oxygen from entering the medium, and as the fermentation progressed, the viscosity of the fermentation broth increased, which could further prevent air from entering the medium. In the agitated culture, the entry of oxygen was accelerated, so that the dissolved oxygen in the medium was higher, and the productivity of BNC in agitated culture was higher than that in the static culture [10]. Although the BNC productivity could be improved in the agitated culture, the production was often reduced. A large amount of glucose was oxidized to gluconic acid due to the excessive oxygen, and the shear stress from the agitated cultivation disturbed the synthesis of BNC [11].

The molecular structure of BNC produced from the agitated culture was similar to that from the static culture, but the morphology was different. Under agitated culture conditions, bacterial nanocellulose was produced as a well-dispersed slurry as irregular masses such as granule, stellate, and bundle strand, while BNC produced from the static culture existed in the form of hydrogel film [12]. In static culture, BNC formed a dense mesh membrane, which had higher crystallinity, polymerization degree and Young's modulus than BNC produced in the agitated culture. However, the water holding capacity of the BNC produced in the agitated culture was relatively high [12]. These may also be caused by sheer stress.

Due to the large shear stress caused by agitated culture, the stability of microfilament entangled to form microwire bundles was reduced during the synthesis and assembly of BNC, thus reduced the production of BNC [13]. The addition of polyglutamic acid (PGA) might reduce the interference caused by the shear stress. And PGA is a non-toxic, recyclable environmentally friendly material.

γ-Polyglutamic acid (γ-PGA) is a kind of polymer bond formed by the condensation of L-glutamic acid or D-glutamic acid monomers through α-amino and γ-carboxyl bonds through microbial fermentation. It was also an environmental-friendly polymer with strong water absorption, water retention, good biodegradability, and strong adsorption [14]. It had been widely used in daily chemical, light industry, medicine, food, agriculture for improving crop production, and many other fields [15–18].

There were studies on techniques improving BNC production. Most of the previous studies were carried out to improve the production by screening strains and adding certain chemicals into the culture medium. A certain concentration of ethanol was added to improve BNC production from fructose, and the ATP content, cell concentration, and fructose consumption rate of living cells were improved [19]. There were also other researchers improved BNC production by adding chemicals or polymers to the medium, including acetic acid, lactic acid, glucuronic acid oligosaccharides (SSGO), and carboxymethyl cellulose (CMC) [20–24]. It was reported that the addition of 0.8% and 1.5% CMC (w/v) improved the BNC production [20]. When acetic acid was added to the medium, it also improved the fermentation of BC, by reducing pH and aggravating glucose decomposition [20]. So far, the effects of those additives were only studied under static culture conditions. A few studies investigated the BNC production in agitated culture. There were a few researchers stating that addition of water soluble polymers into the medium improved production of fermentation products, although the mechanism was still unknown [25]. This study will focus on the effect of adding a small amount of novel polymer on the BNC production in agitated culture.

The purpose of this study was to investigate the effect of adding a small amount of γ-PGA to the medium on the BNC production in agitated culture, and the optimal concentration of γ-PGA addition was investigated. The improved BNC production under different initial pH conditions were also studied. The physicochemical properties of BNC produced in the culture medium with polyglutamic acid were tested using scanning electron microscopy (FE-SEM), Fourier transform infrared spectroscopy (FTIR), thermogravimetric analysis (TGA) and X-ray diffraction (XRD), and degree of polymerization (DPv) and fiber density were analyzed.

## Results and discussion

### The effect of polyglutamic acid on BNC agitated cultivations

**Fig. 1:**
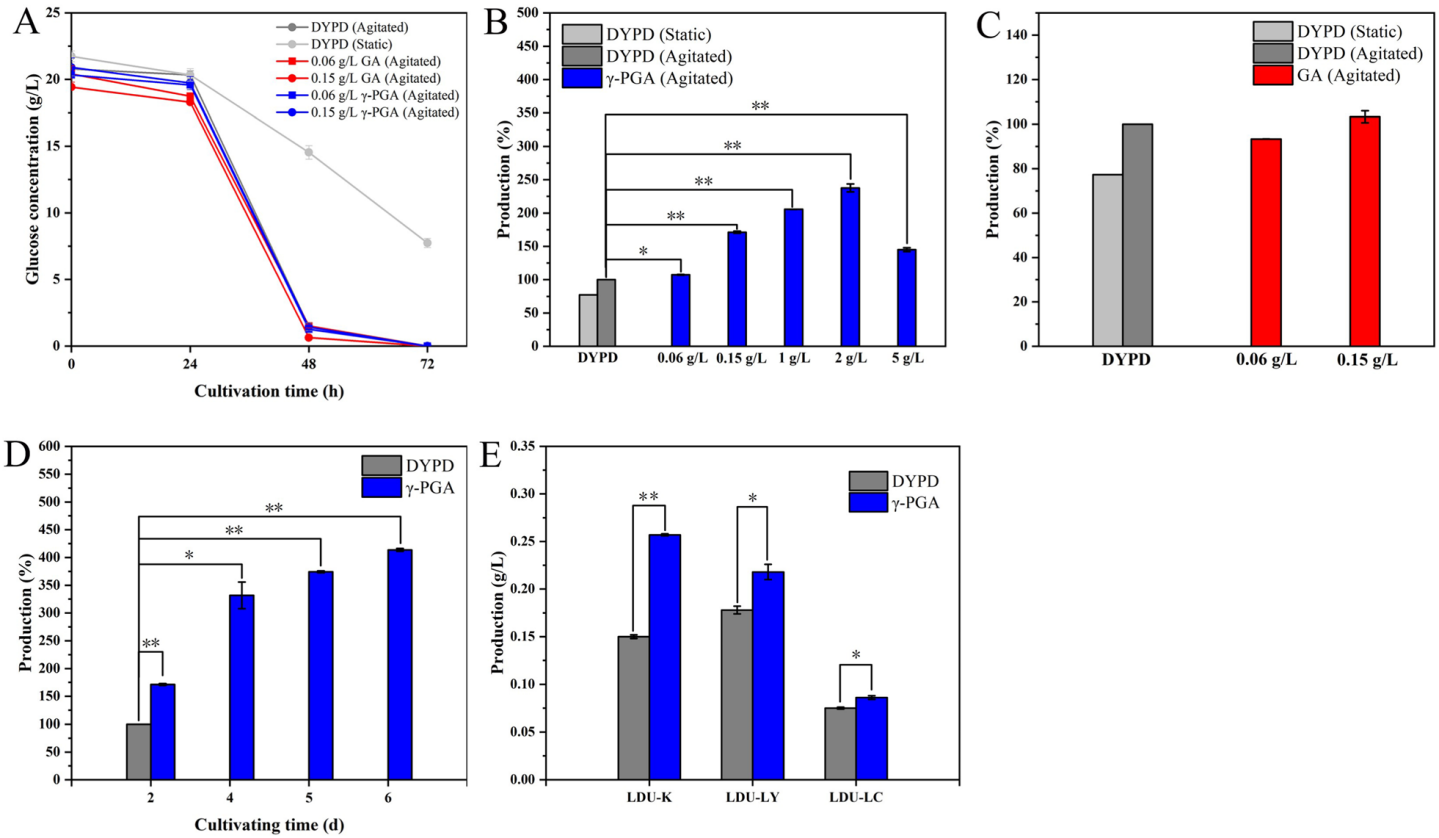
Glucose consumption and BNC production from the medium with γ-polyglutamic acid (γ-PGA), glutamic acid (GA), and the DYPD medium (diluted YPD medium). **A** The glucose concentration of medium with γ-PGA (blue), GA (red), DYPD agitated (dark gray), and DYPD static (French gray), glucose initial concentration of 20 g/L, initial pH 5.0. **B** and** C** The BNC production (%) from medium with γ-PGA (blue), the DYPD agitated fermentation (dark gray), and the DYPD static fermentation (French gray). **D** The BNC production (%) from the DYPD medium and the medium with γ-PGA (blue) after cultivation for 2, 4, 5, 6 days. **E** The BNC production using three different strains: LDU-LC, LDU-LY*,* and LDU-K. *Student’s t test, *P* < 0.05 **Student’s t test, *P* < 0.01

The residual glucose in the BNC fermentation with/without γ-PGA and glutamic acid (GA) is shown in Fig. 1. The results showed that the fastest rate of glucose consumption appeared between 24 and 48 h, and about 84.3% to 91.7% of the total glucose was consumed. The slower glucose consumption appeared between 0 and 24 h and 48 to 72 h, and the glucose was fully consumed before about 72 h (Fig. 1A). Due to the inability to supply oxygen in time, it generally took 6–20 d in static culture before the glucose was consumed [26]. And the agitated culture apparently accelerated the consumption rate of glucose and the fermentation process, and successfully reduced the time for the fermentation to 3 days.

### BNC production

Experiments were carried out to determine the optimal concentration of γ-PGA for improving the BNC production, and γ-PGA concentrations of 0.06 g/L, 0.15 g/L, 1 g/L, 2 g/L, and 5 g/L were examined (Fig. 1B). The BNC production from the DYPD medium (diluted YPD medium) was used as the control. The BNC production from the γ-PGA medium was significantly higher than that from the DYPD medium. BNC production from the 2 g/L γ-PGA medium was the highest, which was 141.37% higher than that from the DYPD medium. However, the BNC production decreased as the γ-PGA addition increased to 5 g/L. The concentration of 2 g/L γ-PGA was the optimal concentration, and 5 g/L γ-PGA seemed began to inhibit the BNC production (Fig. 1B). The BNC production from the medium with 0.15 g/L γ-PGA and 0.06 g/L γ-PGA was 66.7% and 7.3% higher than that from the DYPD medium, respectively (Fig. 1B). The medium added with the corresponding concentrations of glutamic acid were used as control culture medium, respectively, and the BNC production from the γ-PGA medium was significantly higher than those from the glutamic acid medium (Fig. 1C). The results indicated that the positive effects of γ-PGA on BNC production might not be because that γ-PGA was used as nitrogen source. The BNC fermentation under the agitated conditions was continued until the 6th d, and the BNC production gradually increased before the end of the 6th d (Fig. 1D). The results indicated that the BNC seemed stable and did not degrade, three different strains were studied, and the positive effects of γ-PGA on BNC production occurred for all the three bacteria strains, LDU-LC (*Komagataeibacter rhaeticus*), LDU-LY (*Komagataeibacter sp*.), and LDU-K (*Komagataeibacter intermedius*) (Fig. 1E).

The BNC yield from the γ-PGA medium was higher than those from the glutamic acid medium and DYPD medium. The BNC yield from the 0.15 g/L γ-PGA medium was the highest, and the corresponding productivity reached 0.1285 ± 0.0005 g/(L × d), which was 71.3% higher than that from the DYPD medium (Table 1). The conversion rate of glucose to BNC in the 0.15 g/L γ-PGA medium was the highest. The conversion rate of the initial glucose to BNC and the conversion rate of the consumed glucose to BNC in the 0.15 g/L γ-PGA medium were 70.8% and 69.2% higher than those from the DYPD medium, respectively. As the addition of polyglutamic acid increased from 0.06 g/L to 0.15 g/L, the BNC yield increased from 0.161 g/L to 0.257 g/L, accordingly (Table 1). Although the production from the 0.15 g/L γ-PGA medium was lower than that from the 2 g/L γ-PGA medium, a much smaller amount of γ-PGA was used, making the cost from γ-PGA more economic favorable. Therefore, γ-PGA at a concentration of 0.015% (w/v) was used for subsequent experiments.

**Table 1:** Glucose consumption rate, BNC productivity and production (P, product) based on initial and consumed glucose

Groups^a^	Culture condition	Yp (g/L)	BNC production rate [g/(L × d)]	Glucose consumption rate [g/ (L × d)]	YP/initial Glc(g/g)	YP/consumed Glc (g/g)
DYPD	Agitated	0.1500 ± 0.002	0.0750 ± 0.0010	9.6700 ± 0.4100	0.0072 ± 0.0001	0.0078 ± 0.0001
	Static	0.0780 ± 0.002	0.0390 ± 0.0010	6.9980 ± 0.1728	0.0109 ± 0.0005	0.0169 ± 0.0007
γ-PGA	0.06 g/L	0.1610 ± 0.001^∗^	0.0805 ± 0.0005	9.5400 ± 0.0000	0.0079 ± 0.0000	0.0084 ± 0.0001
	0.15 g/L	0.2570 ± 0.001^∗∗^	0.1285 ± 0.0005	9.7400 ± 0.3900	0.0123 ± 0.0000	0.0132 ± 0.0001
GA	0.06 g/L	0.1400 ± 0.002	0.0700 ± 0.0010	9.4560 ± 0.3030	0.0069 ± 0.0001	0.0074 ± 0.0001
	0.15 g/L	0.1520 ± 0.002	0.0760 ± 0.0010	9.4000 ± 0.0400	0.0078 ± 0.0001	0.0081 ± 0.0001

^a^Results based on 48 h old cultures

^*^P < 0.05 with Student's t test, **P < 0.01 with Student's t test. Glucose consumption rate

Experiments show that low concentration of γ-PGA had a significant effect on increasing the production of BNC, and also suitable for a variety of BNC-producing strains. However, the current exploration was only based on 50 mL, which can be extended to larger systems and fermentation tanks in the future.

### BNC fermentation with different initial pH

**Fig. 2:**
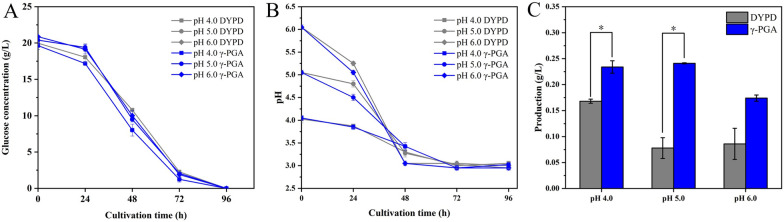
BNC fermentation with different initial pH in the medium with γ-PGA (blue) and DYPD (gray). **A** Glucose consumption and **B** pH change in the medium with γ-PGA (blue) and DYPD (gray) at different initial pH values for every 24 h. **C** The production of BNC after 96 h under different initial pH conditions from the medium with γ-PGA (blue) and DYPD (gray). The initial concentration of glucose was 20 g/L, and0.15 g/L γ-PGA was added. *P < 0.05 with Student's t test

Under the culture conditions of 120 rpm and 30℃, the pH values of the three experiments all decreased during the fermentations (Fig. 2B). The pH in all experiments decreased to 3 in 72 h and remained stable until the end of the fermentation (Fig. 2B). Glucose consumption in the three experiments with different initial pH is shown in Fig. 2A. Due to the initial medium configuration and the random error of the tester, the initial glucose concentration of different experiments was slightly different. All experiments showed fast glucose consumption rate between 24 and 72 h, and the consumption was about 36.3% between 24 and 48 h and 48.6% between 48 and 72 h, respectively. The glucose consumption was relatively slower during 0–24 h and 72–96 h, and the glucose was almost completely consumed at about 96 h (Fig. 2A).

During the agitated cultivations, the BNC production from the γ-PGA medium was higher than that from the DYPD medium in all the three experiments with different initial pH. The BNC production with initial pH 5.0 from the DYPD medium was the lowest, and was only 0.078 g/L, and the BNC production from the γ-PGA medium with initial pH 5.0 was the highest, and reached 0.241 g/L (Fig. 2C), and the difference of BNC production from the γ-PGA medium and the DYPD medium was significant, (*p* < 0.05, Table 2). The improvement of the BNC production was about 209%. As for the experiments with initial pH of 4.0, the BNC production from the γ-PGA medium was improved for about 39.3%, compared with that from the DYPD medium. The production from the γ-PGA medium with initial pH of 6.0 was improved for about 102.3%, compared with that from the corresponding DYPD medium (Fig. 2C). The results showed that pH 5.0 was the optimal initial pH. Comparing the two experiments of pH 4.0 and pH 5.0, it was found that the BNC production from the 0.15 g/L γ-PGA medium was more stable than those from the DYPD medium.

**Table 2:** Glucose consumption rate, BNC productivity, and BNC production (P, product) based on initial and consumed glucose

Groups^a^	pH values	Yp(g/L)	BNC production rate [g/ (L × d)]	Glucose consumption rate [g/ (L × d)]	Y_P/initial Glc_ (g/g)	Y_P/consumed Glc_(g/g)
DYPD	pH 4.0	0.1680 ± 0.0040	0.0420 ± 0.0010	4.9925 ± 0.0575	0.0084 ± 0.0002	0.0084 ± 0.0002
	pH 5.0	0.0780 ± 0.0200	0.0190 ± 0.0050	4.9100 ± 0.1500	0.0040 ± 0.0010	0.0040 ± 0.0010
	pH 6.0	0.0860 ± 0.0300	0.0215 ± 0.0075	5.2175 ± 0.0350	0.0041 ± 0.0014	0.0041 ± 0.0014
γ-PGA	pH 4.0	0.2340 ± 0.0120*	0.0585 ± 0.0030	5.1400 ± 0.0225	0.0114 ± 0.0006	0.0114 ± 0.0006
	pH 5.0	0.2410 ± 0.0010*	0.0603 ± 0.0025	5.1575 ± 0.0575	0.0117 ± 0.0000	0.0117 ± 0.0000
	pH 6.0	0.1740 ± 0.0060	0.0435 ± 0.0015	5.0951 ± 0.1150	0.0085 ± 0.0003	0.0085 ± 0.0003

^a^Results based on 96 h old cultures. The value of Glucose consumption rate was calculated based on the results of 48 h (2 d)

^*^P < 0.05 with Student’s t test, **P < 0.01 with Student's t test

Among the six experiments, the difference between the γ-PGA medium pH 5.0 experiment and DYPD medium pH 5.0 experiment was the most obvious, and the BNC production rate of the γ-PGA experiment was 0.0603 ± 0.0025, and was 217.4% higher than that of the DYPD medium pH 5.0 experiment. The consumption rate of glucose was slightly higher than that in the DYPD medium for 5.0%. In the DYPD medium, the productivity and glucose consumption rate were the highest at pH 4.0, which was similar to the result of Chen et al. [11]. The higher productivity in their study may be due to the higher glucose content in the medium (25 g/L, compared to 20 g/L in this study), lower rotation speed (100 rpm, compared to 120 rpm in this study), and different bacteria strains. However, the conversion rate of glucose to BNC in the γ-PGA medium pH 5.0 experiment was the highest, and was 192.5% higher than that in the DYPD medium pH 5.0 experiment. Since glucose was completely consumed at the end of this experiment, the conversion rates of initial glucose and consumed glucose to BNC were the same.

According to the experiment, the addition of γ-PGA might help with the rapidly decline of pH, which was favorable to the BNC-producing bacteria, promoted BNC synthesis, and improved the BNC production.

### BNC analytical studies

**Fig. 3:**
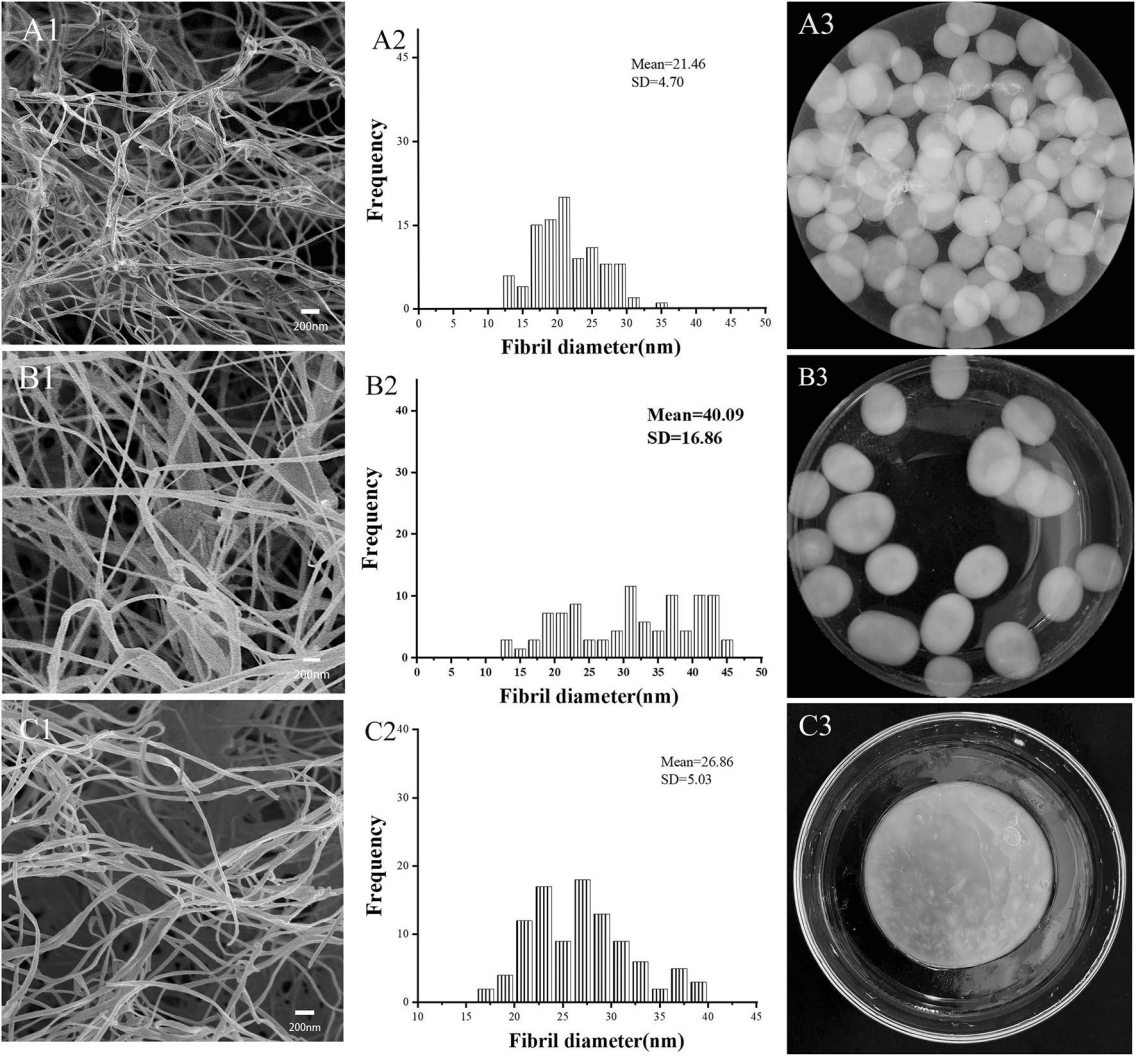
FE-SEM images and fiber diameters of BNC prepared from the DYPD agitated culture (**A1**, **A2**) and static culture (**C1**, **C2**), the medium with γ-PGA (**B1**, **B2**). Amplification: **A1**–**B1**, **A2**–**B2**, **C1**–**C2**, × 10,000. BNC production diagram of BNC prepared by DYPD agitated culture (**A3**) and static culture (**C3**), and the medium with γ-PGA (**B3**). Fiber diameters were calculated through the ImageJ software analysis

The surface morphology of BNC was tested using field FE-SEM, and the results are shown in Fig. 3. The BNC produced from the agitated culture medium supplemented with γ-PGA and DYPD had a similar appearance, showing a gel-like balls floating on the surface of the medium. The fibrils form a three-dimensional network structure and were basically uniform in shape. The diameters of BNC fibers prepared from DYPD medium agitated culture were 12.5–35.0 nm (Fig. 3A2) and were 16.5–40.0 nm from the static culture (Fig. 3C3), while the diameters of BNC fibers prepared from the medium adding γ-PGA were 12.5–45.0 nm (Fig. 3B2). The average diameter of BNC fibers prepared from the DYPD medium in the static culture was thicker than that from the agitated culture (Fig. 3A1, A2 and C1, C2). And the average diameter of BNC fibers prepared from the γ-PGA medium was about twice thicker than that of BNC fibers prepared from the DYPD medium (Fig. 3A1, A2 and B1, B2). It was found that the BNC hydrogel pellets from the DYPD medium were more transparent than those from the γ-PGA medium (Fig. 3A3 and B3), indicating that the BNC fibers produced from the γ-PGA medium were thicker and denser. That might be related to the relief of γ-PGA on the mechanical force from the agitating. BNC with improved fiber diameter and density could be potentially used to manufacture building materials, such as cement asbestos board and glass fiber reinforced plastics.

Zhang [27] measured the BNC produced by *Acetobacter xylinum* using an atomic force microscope, and the average diameter of the BC fibrils was 24.1 ± 0.5 nm, which was similar to that from the control DYPD medium in this study. BNC microprotofibers with diameters ranging from approximately 24.0–55.0 nm was produced under dynamic culture conditions in a previous study [28]. Similarly, Khan et al. [29] and Abol-Fotouh et al. [30] reported BNC nanofiber diameters ranging from 10 to 90 nm using fig extracts supplemented with HS medium. Overall, the BNC fiber diameters from this study fell within the range reported by other researchers.

**Fig. 4:**
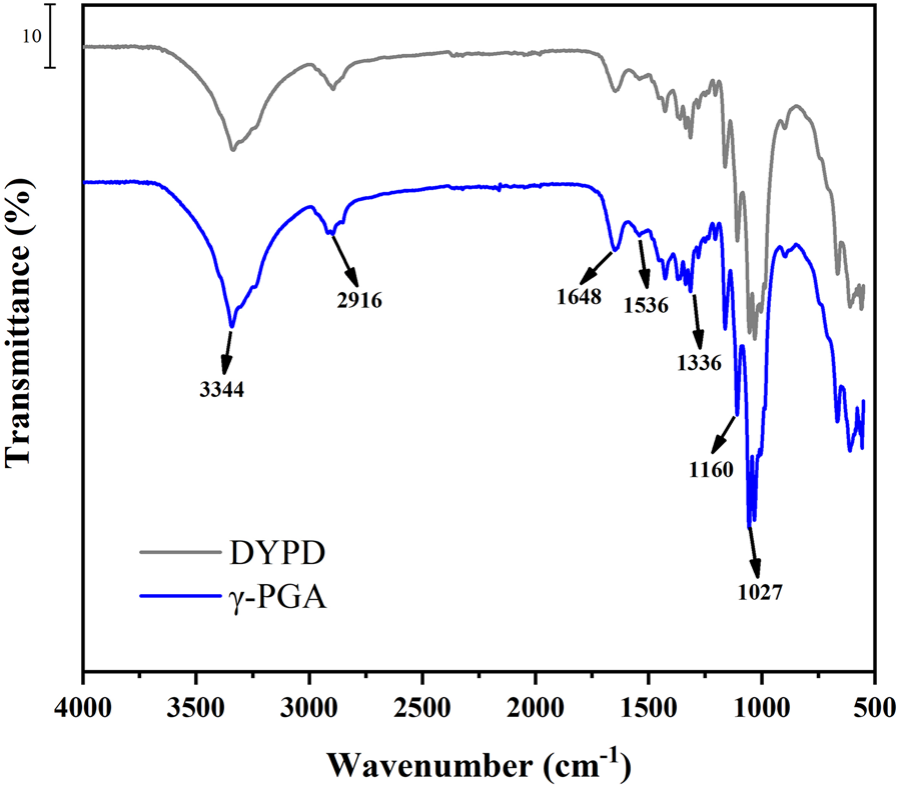
FTIR spectra of BNC prepared from the DYPD (gray) and medium with γ-PGA (blue)

FTIR spectra of BNC prepared from the γ-PGA medium and DYPD showed similar characteristic peaks (Fig. 4). The peak at 1027 cm^−1^ was characteristic of the glycosidic bond of cellulose, while the peak at 1160 cm^−1^ corresponded to the antisymmetric C–O–C stretching of cellulose [31]. The peak at 1336 cm^−1^ was attributed to the C–H out-of-plane bending vibration, while the peak at 1536 cm^−1^ corresponded to the -NH shear vibration. Additionally, the peak at 1648 cm^−1^ could be attributed to the C = O-stretching vibration, while the peaks at 2916 cm^−1^corresponded to the C–H-stretching vibration in cellulose. Furthermore, the peak at 3344 cm^−1^ could be attributed to the OH vibration in cellulose [32].

The results confirmed that the samples were composed of cellulose, as evidenced by FTIR spectroscopy. There were no significant differences observed between the two different BNC preparations, indicating that they had almost identical chemical structure and exhibited the unique characteristics of cellulose [33]. These findings were roughly similar to previous research results, demonstrating the chemical stability and homogeneity of BNC, which was an ideal trait for its various applications in biomaterials and biotechnology.

**Fig. 5:**
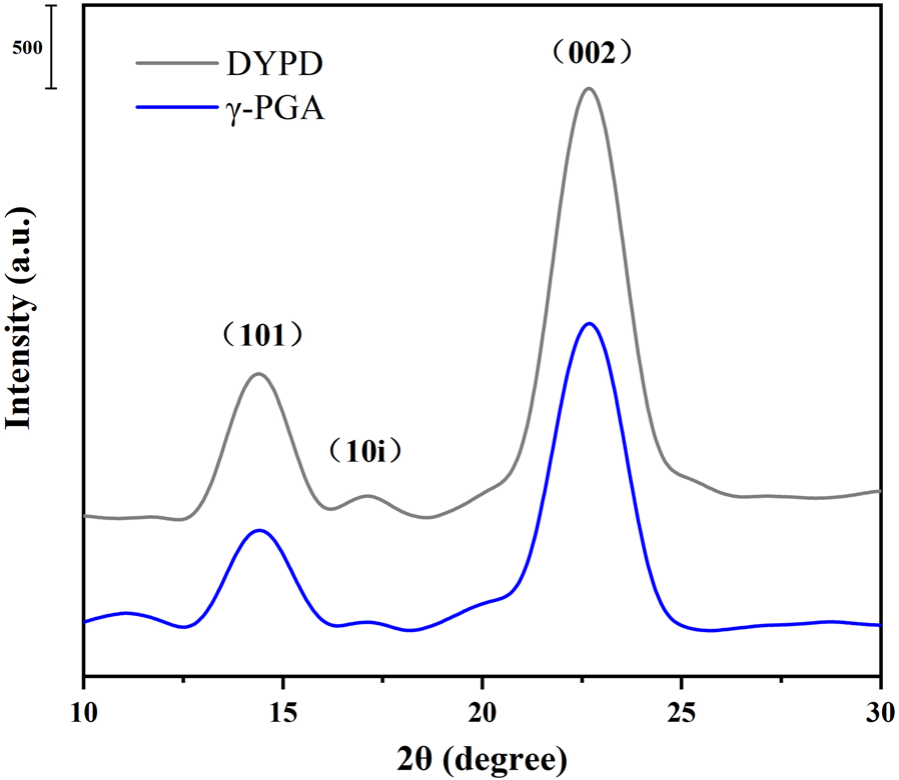
XRD patterns of BNC produced by DYPD (gray) and medium with γ-PGA (blue)

The X-ray diffraction (XRD) analysis was conducted. The results are presented in Fig. 5, which depicted three distinct peaks observed at 2θ = 14.3°, 16.5°, and 22.5°. These peaks corresponded to the diffractions of the (101), (10i), and (002) crystallographic planes of the cellulose structure, as determined using the XRD peak-fitting method. Further analysis was undertaken to determine the interplanar spacing (d-spacing), size of each crystalline plane (ACS), and peak broadening (FWHM). The analysis ultimately facilitated the calculation of the crystallinity index (CrI) and the identification of the primary cellulose phase (Iα or Iβ). The results are shown in Table 3. The crystallinity of BNC produced from the γ-PGA medium was higher (82.95%) than that of the BNC produced from the DYPD medium (78.65%). It might be related to that γ-PGA can help the crystal formation (folding or assembly of cellulose). Due to the reduction of the shear stress, γ-PGA could potentially provide a more stable environment for the folding and crystal formation of cellulose molecules, and increased the crystallinity. The fiber with high crystallinity often has stronger mechanical properties and corrosion resistance. The BNC with higher crystallinity could be potentially used as a fabric coating agent for functional coating of the fabric and a finishing agent for functional finishing of the fabric.

**Table 3:** Crystallinity of BNC produced from the DYPD medium (gray) and the medium with γ-PGA

Matrices	d-spacing (nm)			ACS (nm)			FWHM			CrI (%)	DA	Difference in Bragg angle (degrees)
	(101)	(10i)	(002)	(101)	(10i)	(002)	(101)	(10i)	(002)			
DYPD	0.61	0.52	0.39	7.71	7.19	4.70	1.10	0.86	1.33	78.65	Iα	2.31
γ-PGA	0.61	0.52	0.39	4.58	6.53	3.53	1.30	0.92	1.68	82.95	Iα	2.44

**Fig. 6:**
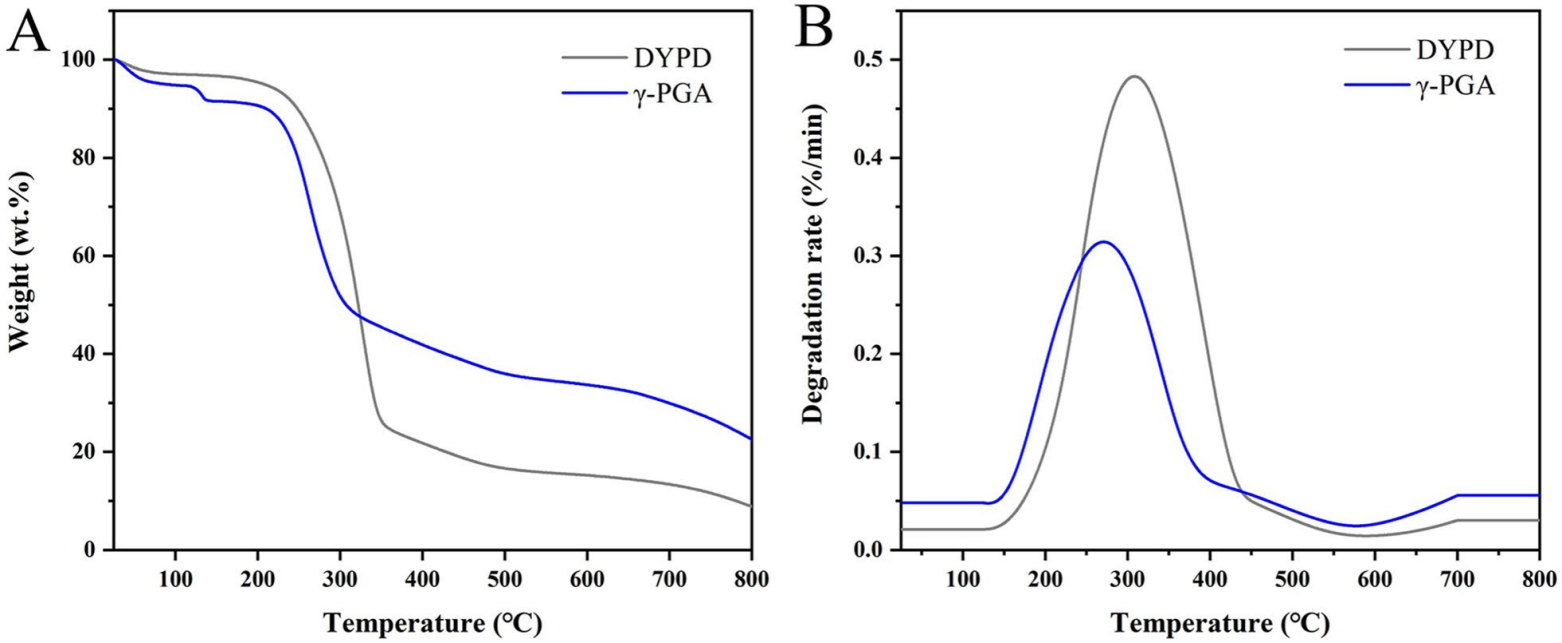
**A** Thermogravimetric (TG) and **B** differential thermogravimetric (DTG) curves of BNC prepared from the DYPD (gray) and medium with γ-PGA (blue)

Thermogravimetric analysis was conducted on the BNC prepared from the γ-PGA medium and the DYPD medium. The results showed that all BNC materials showed similar thermal degradation temperatures, and the overall degradation trends were similar. However, it could be seen that BNC produced from the medium with γ-PGA had higher residual weight than that from the DYPD medium, and the rate of the weight loss was slower in the stage of the rapid weight decline, so the BNC produced from the medium with γ-PGA had higher thermal stability (Fig. 6). That might also be related to the higher crystallinity of the BNC produced from the γ-PGA medium. The temperature range for degradation rates greater than 0.2 (µg/min^−1^) was found to be 232–358 ℃. However, when the degradation rate was larger than 0.5%/min, the differences between the BNC produced from the medium with γ-PGA and DYPD became larger, and the temperature range was 255–288 ℃ for the BNC produced from the medium with γ-PGA, while it was 287–350 ℃ for that from the DYPD medium (Table 4). The BNC produced from the medium with γ-PGA curve exhibited two successive decreasing trending sections, with the temperature interval having the fastest degradation rate (> 2.5 µg/min^−1^) showing a sharp weight loss of 50–67%, primarily due to cellulose degradation and some breakdown of glycosidic bonds [34, 35]. This was followed by cellulose carbonation and oxidation, which occurred in the temperature range of 426–700 ℃. These findings provided important insights into the thermal stability and behavior of BNC materials, which informed their use in a variety of industrial applications.

**Table 4:** Thermal degradation properties of BNC produced using DYPD and medium with γ-PGA

Culture medium	DTG peak (℃)	Degradation rate at DTG peak (%/min)	Degradation rate > 0.2 (%/min)	Degradation rate > 0.5 (%/min)
DYPD	330	0.66	252–358	287–350
γ-PGA	268	1.23	232–319	255–288

The DPv values from the medium with γ-PGA were significantly higher than from the DYPD medium, and the fiber density of the BNC produced from the medium with γ-PGA was 67.4% higher than that from the DYPD medium. The results indicated that addition of γ-PGA not only improved the degree of polymerization of BNC, but also improved the fiber density in the 3D nanofiber network of BNC (Table 5). The higher degree of polymerization of the BNC from the medium with γ-PGA might be related to the reason of its low DTG temperature (Table 4), improving the thermal stability of BNC. The DPv values of the BNC produced from the medium with γ-PGA in this study was 4244.0 ± 324.0, which was higher than the DPv values previously reported [36].

**Table 5:** Viscometric degree of polymerization (DPv) and fiber density of BNC produced using the DYPD medium and the medium with γ-PGA

Culture medium	DPv	Fiber density (g/m^3^)
DYPD	3191.0 ± 81.0	3380.5 ± 37.5
γ-PGA	4244.0 ± 324.0	5659.0 ± 37.0

The synthesis of BNC was catalyzed by cellulose synthase in *Acetobacter xylinum*. Cellulose synthase and other proteins constituted the terminal complex (TC), and normal arrangement of the linear terminal complexes appeared as a single row along the longitudinal axis of the cell, and the disruption in cellulose synthesis occurred at the linear TC orientation level of organization [37]. During the synthesis and assembly of BNC, due to the specific arrangement of TC, microfibrils were further entangled to form microfibril bundles [13]. It seemed that the larger shear stress would affect the stability of the arrangement of TC, and the addition of polyglutamic acid may reduce the interference caused by the shear stress.

In this study, addition of γ-PGA was found to improve not only the production but also the quality of BNC, and the fiber diameter, the degree of polymerization, and the fiber density were all improved with the addition of γ-PGA.

## Conclusions

The glucose consumption rate and production of BNC were higher under agitated culture conditions, but glucose conversion rate was often lower due to the quick oxidation of glucose. In this experiment, it was found that addition of trace amount of γ-PGA could significantly increase the glucose conversion rate and BNC production in the early stage of the fermentation. It is the first time reported that using polyglutamic acid could improve the production of bacterial nanocellulose. The BNC production from the 0.15 g/L γ-PGA medium was significantly improved compared to that from the DYPD medium. BNC was analyzed using FTIR, TGA, the chemical composition of the BNC produced from the γ-PGA medium and DYPD medium were found similar. However, the average diameter of BNC fibers prepared from the medium added with γ-PGA was twice thicker than that of BNC fibers prepared from the DYPD medium; according to the FE-SEM analysis, the XRD analysis showed that the crystallinity of BNC produced from the γ-PGA medium was higher than that produced from the DYPD medium. The thermogravimetric analysis also revealed that BNC produced from the medium with γ-PGA had higher thermal stability than that from the DYPD medium, and the degree of polymerization (DPv) of the BNC produced from the medium with γ-PGA was significantly higher than that from the DYPD medium. That might be related to the relief of polyglutamic acid on the mechanical force from the agitating. As the BNC produced from the γ-PGA medium had superior properties, it had better potential to be used as a fabric coating agent for functional coating of fabrics or a finishing agent for functional finishing of fabrics (shape memory function). In this study, by adding the additive γ-PGA to the medium, the conversion rate of glucose to BNC was increased under the condition of agitating culture, which laid a solid foundation for larger agitating production such as airlift and agitated tank BNC agitating culture in the future. The effect of γ-PGA addition in the bioreactor experiments needs further investigation.

## Materials and methods

### Materials

Glucose, peptone, yeast extract, sodium hydroxide, and concentrated sulfuric acid were purchased from Sinopharm Chemical Reagent Co. Ltd. γ-PGA medium was obtained from the College of Agriculture, Ludong University.

### Culture medium and bacterium strain

The medium contained 20 g/L glucose, 5 g/L peptone, and 3 g/L yeast powder as named DYPD medium and was used for seed culture and as control in the experiments. 30 g/L polyglutamic acid solution, 30 g/L-glutamic acid solution. 100 ml shake flask was used, with 50 ml medium in each shake flask. The medium and solution were sterilized at 121℃ for 20 min, and then cooled to room temperature before used. The fermentation medium was prepared by adding in DYPD with a final concentration of 0.06 g/L, 0.15 g/L, 1 g/L, 2 g/L or 5 g/L polyglutamic acid, respectively. Glutamic acid with a concentration of 0.06 g/L or 0.15 g/L were used as controls. The pH was adjusted to 5.0 after the sterilization. In the experiments with different starting pH, the initial pH of the culture medium were adjusted to 4.0, 5.0, and 6.0, respectively, using 20% H_2_SO_4_ aqueous solution and 8 mol/L NaOH. The medium was sterilized at 121 ℃ for 20 min before used.

The main bacterium strain used in this experiment was LDU-K, LDU-LC, and LDU-LY were used as controls, which were previously isolated in our laboratory, and the polyglutamic acid was fermented in our laboratory.

### BNC fermentation

The 100 mL medium shake flask was placed in a fixed-axis shaker, and the agitated culture was carried out at 120 rpm and 30 ℃.

A small amount of fermentation broth was taken out from each shake flask of culture medium, and the initial pH value was measured using pH meter, and the initial glucose concentration was measured. The sugar content and pH were measured every 24 h for 4 days. Calibration curves were made for accurate glucose measurements. The experiments were done in duplicates.

After 96 h of fermentation, the fermentation broth was filtered using a double-layer filter, and the BNC was collected and thoroughly washed using sterile water. The wet BNC was dried at 60 ℃ for 6 h, and was weighed using a standard weighing balance for production statistics.

## BNC characterization

### Scanning electron microscopy analysis

The washed and dried BNC samples were fixed and gold plated for FE-SEM imaging. The morphology and structure of the prepared biological nanomaterials were determined by high-resolution field emission transmission electron microscopy (FE-TEM, su8010, Hitachi).

### Fourier infrared spectroscopy analysis

The BNC samples were subjected to Perkin Elmer FTIR spectrophotometer (Thermo Nicolet 6700, NEXUS, TM) equipped with an attenuated total reflection (ATR) assembly with a zinc selenide (ZnSe) crystal. Sixteen scans of each sample within a range from 4000 to 500 cm^−1^ at a 4 cm^−1^ resolution were collected.

### XRD analysis

BNC was analyzed using a Rigaku Smart LabSEx X-ray diffractometer (XRD) from Rigaku, Japan. This instrument employs CuKα1 radiation (*λ* = 0.154178 nm). X-ray diffraction data were collected in the 2θ range of 5° to 90°, with a step size of 0.02° and a scanning rate of 10°/min. Jade software was utilized for phase identification and data analysis, referencing the diffraction patterns provided by the International Center for Diffraction Data (ICDD).

### Thermogravimetric analysis

Thermogravimetric analysis (TGA) was carried out on the TGA2 model weight analyzer produced by METTLER. 3–5 mg of the BNC sample to be tested was weighed and placed in a tray, and was heated from 25 to 800 ℃ under nitrogen environment at a constant heating rate of 10 ℃/min.

### Measurement of DPv and fiber density of BNC

The DPv of the BNC was measured three times with a viscometer, as previously reported [38]. The weight of harvested BNC hydrogels was measured before and after being air-dried, the fiber density was defined as the dry weight divided by the volume of the hydrogel [39].

## Data Availability

Data will be made available from the corresponding author on reasonable request.
